# Seeing Sounds: The Role of Vowels and Consonants in Crossmodal Correspondences

**DOI:** 10.1177/20416695221084724

**Published:** 2022-03-16

**Authors:** Yang-Chen Shen, Yi-Chuan Chen, Pi-Chun Huang

**Affiliations:** Department of Psychology, 34912 National Cheng Kung University, Tainan; Department of Medicine, 145474 Mackay Medical College, New Taipei City; Department of Psychology, National Cheng Kung University, Tainan

**Keywords:** consonants, vowels, bouba–kiki effect, sound shape correspondence

## Abstract

Crossmodal correspondences refer to the fact that certain domains of features in different sensory modalities are associated with each other. Here, we investigated the crossmodal correspondences between speech sounds and visual shapes. Specifically, we tested whether the classification dimensions of English vowels (front–central–back) and consonants (voiced–voiceless, sonorant–obstruent, and stop–continuant) correspond to visual shapes along a bipolar rounded–angular dimension. We adapted eighteen meaningless pseudowords from a previous study that corresponded to either the round or the sharp concept. On each trial, the participants heard one of the pseudowords and saw a rounded shape and an angular shape presented side-by-side on the monitor. Participants judged which shape provided a better match to the spoken pseudoword. A logistic regression was conducted in order to elucidate the effectiveness of classification dimensions of phonemes when predicting variations in the sound–shape matchings. The results demonstrated that the sound–shape matchings were predictable using front–central–back dimensions of vowels, and voiced–voiceless and stop–continuant dimensions of consonants. Hence, we verified that sound–shape matching is underpinned by contrasting dimensions in both vowels and consonants, therefore demonstrating crossmodal correspondences at the phonetic level.

## Introduction

Crossmodal correspondences refer to the associations between some properties in one sensory modality to those in another sensory modality (see Spence, 2011; Spence & Sathian, 2020 for reviews). One of the most classic examples of crossmodal correspondences occurs between nonsense speech sounds and visual shapes where “bouba” and “kiki” are mapped onto rounded and angular shapes, respectively (called the “bouba–kiki effect” hereafter; Köhler, 1929, 1947; Ramachandran & Hubbard, 2001). It has been suggested that the bouba–kiki effect is universal (Bremner et al., 2013; Davis, 1961; Ramachandran & Hubbard, 2005; Rogers & Ross, 1975), and it has been demonstrated in infants and young children (Asano et al., 2015; Imai et al., 2015; Maurer et al., 2006; Ozturk et al., 2013). Furthermore, such sound–shape mappings have been demonstrated to occur automatically and unconsciously (Hung et al., 2017; Parise & Spence, 2012). Although the bouba–kiki effect has been replicated by many research groups (see Ramachandran et al., 2020; Spence & Sathian, 2020 for reviews), there is no consensus regarding the critical properties of phonemes that afford this association with visual properties.

Phonemes contain multidimensional information related to both acoustic and articulatory features. Some researchers have suggested that correspondences between vowels and visual shapes dominate the bouba–kiki effect (Maurer et al., 2006; Ramachandran & Hubbard, 2001; Tarte, 1974, 1982). For example, an extensive body of literature suggests that some vowels such as /a/, /o/, and /u/ are round-sounding, whereas other vowels, such as /e/, and /i/ are sharp-sounding (Kovic et al., 2010; Lockwood & Dingemanse, 2015; Maurer et al., 2006; Remez et al., 2003). However, others suggest that consonants play a more dominant role over vowels (Fort et al., 2015; Nielsen & Rendall, 2011; Ozturk et al., 2013). For example, it has been shown that voiceless consonants (e.g., /p/, /t/, and /k/) are associated with angular shapes, whereas the sonorant consonants (e.g., /l/, and /m/) and voiced consonants (e.g., /b/) are associated with rounded shapes. The last notion suggests that vowels and consonants have distinct but complementary roles in sound-shape mappings (Ahlner & Zlatev, 2010). For example, D’Onofrio (2014) reported that back vowels (/a/ and /u/), voiced consonants (/b/, /d/, and /g/), and labial consonants (/b/ and /p/) are significant predictors of matching a nonsense word to rounded shapes. D’Onofrio's results therefore inspired the current investigation of the relative contributions of consonants and vowels to the bouba–kiki effect.

Previous studies of the bouba–kiki effect often used a small set of meaningless sounds created by combining particular phoneme contrasts into the consonant-vowel-consonant-vowel (CVCV) structure, such as /pipe/ made by combining a voiceless consonant and front vowels, and /buba/ made by combining a voiced consonant and back vowels (D’Onofrio, 2014; Fort et al., 2015; Peiffer-Smadja & Cohen, 2019). However, such CVCV pseudowords are uncommon and unrealistic in the English language. Westbury et al. (2018) used a Markov chaining process to create pseudowords consisting of frequent syllable boundaries that highly resemble real English words. In the current study, we therefore used the pseudowords from Westbury et al.'s published norms, half of which are associated with the round concept and the other half of which are associated with the sharp concept, to investigate the contributions of consonants and vowels to sound–shape correspondences. More specifically, there are three critical differences between the current study and Westbury et al. (2018). First, Westbury et al. presented each pseudoword both visually and auditorily, whereas we merely presented each pseudoword auditorily. This critical difference therefore made it possible for us to assess the contributions of consonants and vowels to crossmodal correspondences while avoiding the confounding effects resulting from visual word forms (see also Cuskley et al., 2017). Second, Westbury et al., asked participants to judge whether each pseudoword was suitable for a particular abstract concept (such as “round”, “small”, or “feminine”), whereas we presented two concrete visual patterns for the participants to match. Our procedure should avoid individual differences in defining features of a concept. Third, and most critically, Westbury et al. focused on the phonetic features, phonemes, letters, and bigrams associated with each concept, whereas we aimed to evaluate the mappings between critical contrasting dimensions of consonants and vowels in speech and the rounded–angular dimension of shapes in vision. We classified the phonemes based on the position of the tongue when producing the vowel (front–central–back) and three contrasting dimensions of consonants (voiced–voiceless, sonorant–obstruent, and stop–continuant) that have been used in previous studies (Ahlner & Zlatev, 2010; Aveyard, 2012; D’Onofrio, 2014; Nielsen & Rendall, 2013; Westbury, 2005) and performed a logistic regression to assess whether and how each dimension predicts the sound–shape matching results.

## Methods

### Participants

Ninety-six participants (mean age = 23.1 years, SD = 4.5 year, age range: 20–34 years, 47 males) who are native Mandarin speakers recruited from National Cheng Kung University were reimbursed in return for their participation. All of the participants were naïve to the purpose of the study and gave their informed consent before the experiment. All of the procedures were carried out in accordance with the Declaration of Helsinki and were approved by the Department of Psychology at National Cheng Kung University.

### Apparatus and Stimuli

Stimuli were presented on a 24-inch LED monitor (Samsung S24E390HL) and controlled by a PC compatible with the Psychophysics Toolbox (Brainard, 1997) in the MATLAB (The Mathworks, Matick, MA, USA) environment. The resolution of the monitor was 1,280 × 720 pixels with a refresh rate of 60 Hz. The auditory stimuli were presented through speakers (Genius SP-U150) that were placed 5 cm to the left and right of the monitor.

**Figure 1.**
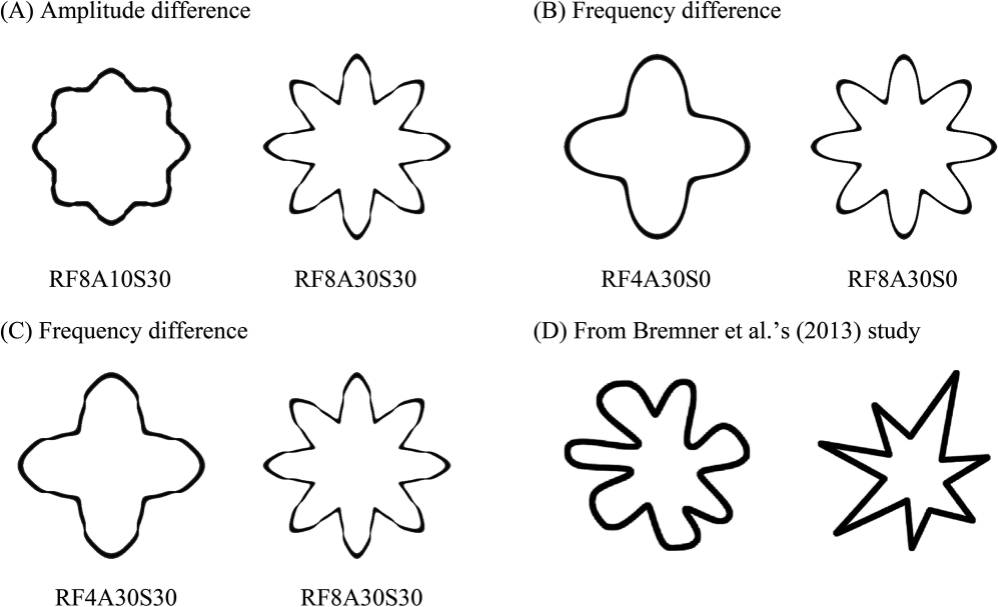
The visual patterns used in the present study. The RF pattern was manipulated in three dimensions: radial frequency (RF, the number of sinusoidal modulations per circle), amplitude (A, the magnitude of the sinusoidal modulations deviating from a circle, ranging from 0 to 100), and spikiness (S, the number of harmonics of triangular wave forms added on top of each sinusoidal modulation). (A) The first pair was RF8A10S30 and RF8A30S30, which differed in amplitude. (B) The second pair was RF4A30S0 and RF8A30S0, which differed in frequency. (C) The third pair was RF4A30S30 and RF8A30S30, which differed in frequency. (D) The visual patterns were the adopted from Bremner et al. (2013). According to Chen et al.’s (2016) results, within each pair, the left pattern was predominantly matched to “bouba”, while the right pattern was predominantly matched to “kiki”.

Four pairs of visual patterns were used. One pair was the patterns commonly-used when testing the bouba–kiki effect in Bremner et al.’s (2013) study, and the other three were radial frequency (RF) patterns that were dominantly matched to “bouba” or “kiki” in Chen et al. (2016; see Figure 1 for the manipulated parameters). Each pattern consisted of a black outline presented against a white background. At a viewing distance of 60 cm, the size of each visual pattern was approximately 13 × 13 degrees. A pair of visual patterns were presented side-by-side on the monitor at the same time (14° of visual angle from center to center).

**Figure 2.**
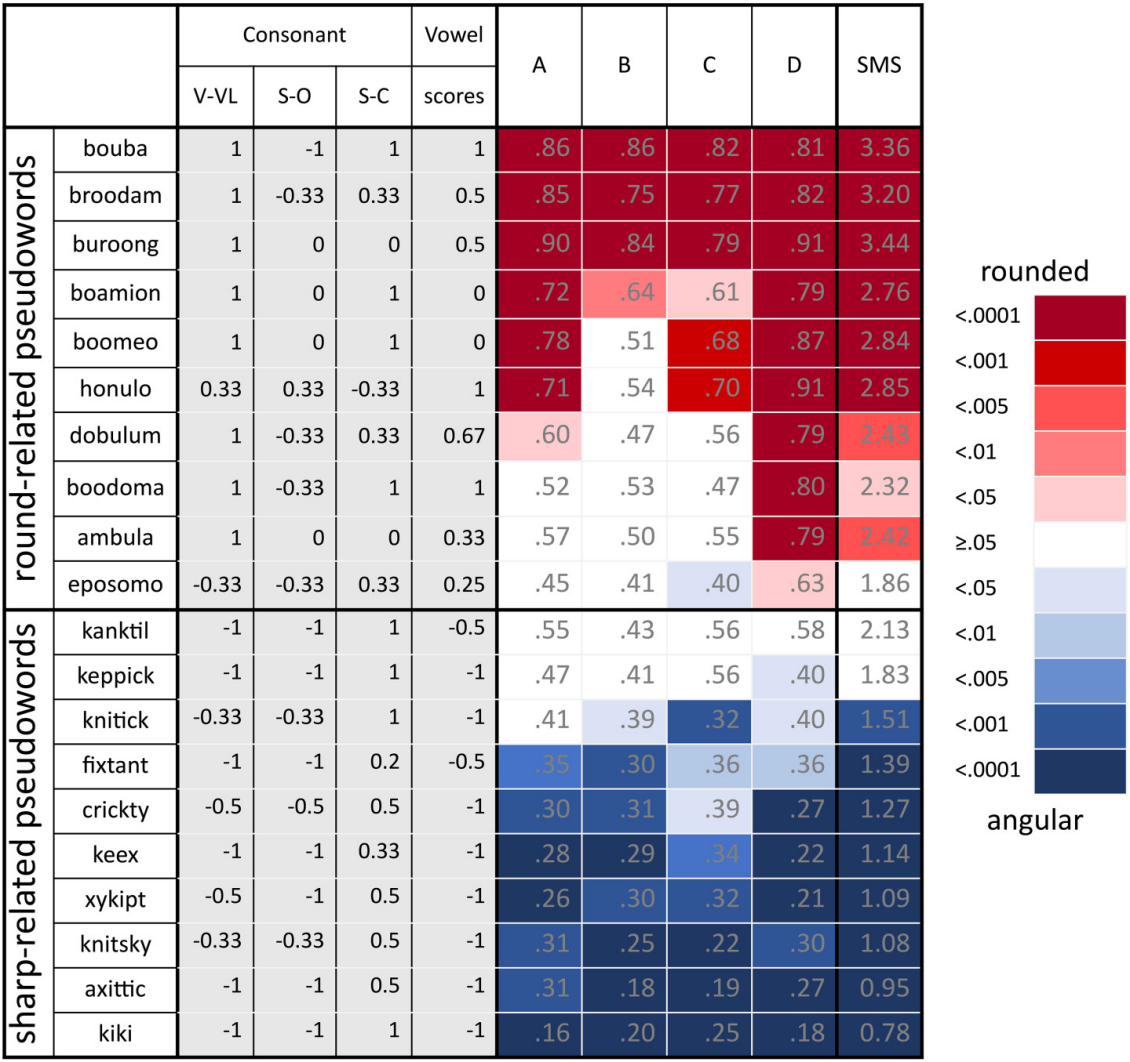
The pseudowords used in the experiment and their matching results. Half of the pseudowords were associated with the round concept and the other half with the sharp concept (Westbury et al., 2018). The normalized proportion scores of each pseudoword in each contrasting dimension are shown, where V–VL represents the voiced–voiceless dimension; S–O represents the sonorant–obstruent dimension; S–C represents the stop–continuant dimension, and the vowel scores are the mean of the tongue position when producing the vowel. The proportion of choosing rounded shape for each pseudoword and each visual-pattern pair (A-D, as shown in Figure 1) are listed in each cell. The pseudowords that were better matched to the rounded shape are represented in red, whereas those better matched to the angular shape are represented in blue. The remaining undetermined matches are represented in white. The color saturation represents the *p* values of the *X*^2^ test for each cell. The rightmost column shows the summed matching score (SMS) and the color saturation represents the *p* values for the one-sample *t*-test.

Eighteen nonsense speech sounds, half of them associated with “round” concept and the other half with the “sharp” concept (corresponding to the “bouba” and “kiki” separation, respectively), were selected from Westbury et al.’s (2018) published norms (see Figure 2). In addition, the sounds “bouba” and “kiki” were tested as well. Each speech sound was produced by a female voice with an English pronunciation developed by Google translator (https://soundoftext.com/). The amplitudes of all sounds were equalized in terms of root-mean-square (RMS) power.

### Design

The consonants and vowels contained in the pseudowords were separated in terms of the following dimensions: For the consonants, we used three classification dimensions of English consonants that were used in previous sound–shape studies, including voiced–voiceless (D’Onofrio, 2014), sonorant–obstruent (Ahlner & Zlatev, 2010; Nielsen & Rendall, 2012, 2013), and stop–continuant (Aveyard, 2012; Westbury, 2005). We characterized the vowel sounds into front, central, and back categories. We calculated the normalized proportion in each dimension of consonants in each pseudoword, respectively. The equation was defined as the number of voiced/sonorant/stop consonants minus the number of voiceless/obstruent/continuant consonants, after which the value was divided by the sum of the number of consonants. Take “bouba” as an example. /b/ is a voiced consonant and there are no voiceless consonants. Thus, the voiced–voiceless normalized proportion is (2 − 0)/(2 + 0) = 1. Consequently, the normalized proportion ranges from −1 to 1, where the 1 indicates that all of the consonants were voiced, sonorant, or stop, whereas the value of −1 indicates that all of the consonants were voiceless, obstruent, or continuant. The pseudowords in the round category have a higher voiced–voiceless normalized ratio than is the case in the sharp category (0.80 ± 0.14 vs. −0.77 ± 0.10, *t*(18) = 9.10, *p* < .001). In addition, the pseudowords belonging to the round category have a higher sonorant–obstruent normalized ratio than those belonging to the sharp category (−0.2 ± 0.11 vs. −0.82 ± 0.09, *t*(18) = 4.18, *p* < .001). However, the pseudowords in the round and sharp category have similar stop–continuant normalized ratios (0.46 ± 0.16 vs. 0.65 ± 0.10, *t*(18) = −1.00, *p* = .17).

In the analysis of the vowels in the pseudowords, we modified the method used in Barton and Halberstadt (2018). Each vowel, according to position of the tongue when producing it (front, central, and back) was assigned an ordinal scale score of −1, 0, or 1. Then, we averaged the position scores so that the scores would also range from −1 to 1. Take “kiki” for example. /i/ is categorized as a front vowel, where the ratio equals [(−1) + (−1)]/2 = −1. The pseudowords in the round category have a higher score for vowels than those in the sharp category (0.53 ± 0.12 vs. −0.90 ± 0.07, *t*(18) = 10.18, *p* < .001).

### Procedure

In each trial, a pair of visual patterns were presented side-by-side on the monitor at the same time, and one of the pseudowords was presented auditorily. The location of each pattern within a pair (left or right) was randomized. The participants had to judge whether the figure presented in the left or right provided a better match to the speech sound. The visual patterns disappeared after the participant pressed a response key. Each participant had to complete 80 trials (20 speech sound × 4 pairs of stimuli) in a completely randomized order. The experiment took about 10 min to complete.

## Results

The proportion of consensual matching to the rounded shape for each pseudoword is shown in each corresponding cell in Figure 2. An *X*^2^ test was conducted to verify any consensual matching between each pseudoword and each pair of visual patterns separately. We also calculated the summed matching score (SMS) for each pseudoword, which was the sum of the matching score for the rounded shape of four pairs of visual patterns. A score of 4 indicates choosing the rounded shape category for every visual pattern pair; a score of 2 represents random choices, and a score of 0 represents choosing the angular shapes for each visual pattern pair. A one-sample *t* test was conducted for the SMS. The results for both the *X*^2^ test and one-sample *t* test (compared to 2) are demonstrated using color code in Figure 2. The pseudowords that were better matched to the rounded shape are represented in red, while those better matched to the angular shape are represented in blue, and those that were undetermined are represented in white. The saturation of the color represents the *p* values of the corresponding test. The results showed that the matchings between pseudowords and shapes were not necessarily consistent across the four pairs of patterns. These variations in the matchings between sounds and shapes made it possible to assess the degree to which the predictabilities of phonemes and their generalizabilities across visual patterns were effective using a logistic regression model.

In order to investigate the contribution of consonants and vowels to the sound–shape matching process, we conducted a logistic regression in the lme4 (linear mixed effect) package (Bates et al., 2015) in R (version 3.2.1). Our hypothesis was that if one of the contrasting phoneme dimensions drives the correspondences of the rounded–angular shapes, then the dimension would be a valid predictor of the sound–shape matching judgments. Thus, the voiced–voiceless, sonorant–obstruent, and stop–continuant ratios of the consonants and the position scores of the vowels were fixed factors (see Methods). The participants and the four pairs of patterns were treated as random factors in the logistic regression model.^1^

The results for fitting are shown in Table 1. When predicting the sound–shape matching judgments, the vowel position score contributed the most (*β* = 0.654 ± 0.062), followed by the voiced–voiceless dimension (*β* = 0.432 ± 0.066). Note that the 95% confidence intervals were overlapped for these two factors, so they should be considered equally effective in predicting the sound–shape matching judgments. The stop–continuant normalized proportion was a weak but still valid predictor (*β* = 0.187 ± 0.074), whereas the sonorant–obstruent dimension was not a valid predictor (*β* = 0.001 ± 0.093).^2^

**Table 1.** Results of the Logistics Linear Regression.

Factors	Coefficient	SE	95% confidence interval		z-values	*p*
			Lower	Upper		
position score of vowels	0.654	0.062	0.533	0.775	10.592	<.001
voiced–voiceless	0.432	0.066	0.303	0.561	6.580	<.001
stop–continuant	0.187	0.074	0.042	0.333	2.519	.012
sonorant–obstruent	0.001	0.093	−0.181	0.184	0.015	.99
Intercept	0.059	0.116	−0.168	0.285	0.509	.61

## Discussion

In order to examine the critical dimensions of phonemes that drive sound–shape correspondences, we adapted pseudowords associated with round or sharp concepts taken from Westbury et al.’s (2018) published norms. The matching results showed that most of the pseudowords matched to corresponding rounded or angular shapes and were consistent with Westbury et al.'s round or sharp concept with three exceptions (eposomo, kanktil, and keppick). More critically, the selected speech sounds that had higher vowel position scores and higher contrast scores in the voiced–voiceless and stop–continuant dimensions of consonants were more likely to be matched to rounded rather than angular shapes. That is, pseudowords containing a higher proportion of the back vowels, and voiced and stop consonants were more likely to be matched to rounded shapes. Interestingly, sonorant–obstruent contrast that significantly differed in the pseudowords associated with the round and sharp concept did not have significant predictive power in the current study. In contrast, the stop–continuant contrast that was similar in the pseudowords associated with the round and sharp concept had weak but significant predictive power. This might also indicate an advantage of using pseudowords that resemble English words in order to reveal the dynamic interactions among phonemes (D’Onofrio, 2014; Fort et al., 2015; Peiffer-Smadja & Cohen, 2019). Note that the current result cannot differentiate whether the sonorant–obstruent contrastive dimension did not contribute to the sound–shape correspondences, or our participants were insensitive to sonorant–obstruent contrastive dimension.

It has been shown that the sound–shape correspondences can be driven by the acoustic properties, and the frontness of the vowels is associated with the second formant in the acoustic properties (Knoeferle et al., 2017). One might therefore attribute the predictive power of phonemes to the range of acoustic differences (i.e., the larger acoustic range of vowels led to their more pronounced predictive power). However, consonants do not have common acoustic properties: the acoustic features of a given consonant depend on the neighboring vowels (e.g., /d/ in /di/ and /du/ are different). Given the larger acoustic variation for vowels than consonants in general, however, the predictive power was similar between vowels and voiced-voiceless consonants in the current study. Thus, the acoustic properties might not be the determinant factor for different predictabilities of vowels and consonants in the sound-shape matching results.

**Figure 3.**
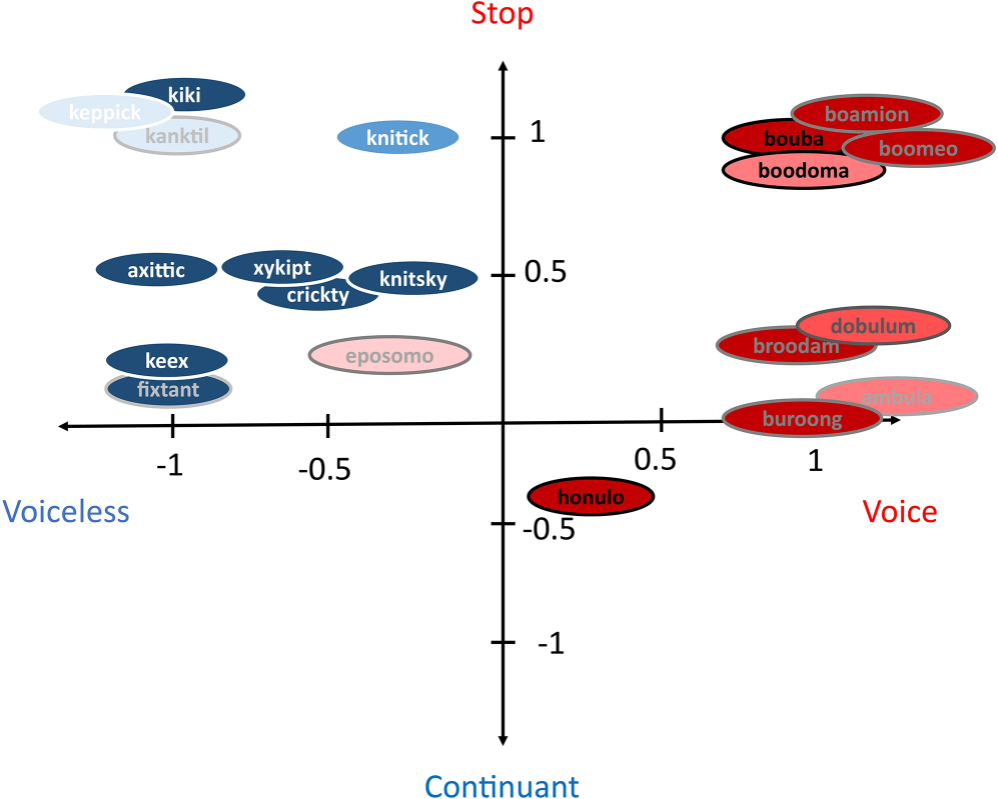
Diagram demonstrating the tested pseudowords on the voiced–voiceless and stop–continuant contrasting dimensions of consonants (the x- and y-axis, respectively). The darkness of the pseudowords and the outlines represents the position scores of the vowels, in which darker color represents higher scores for back vowels, and brighter color represents higher scores for front vowels. The filled color of each ellipse represents the matching consensus to rounded (red) or angular (blue) shapes based on the results in Figure 2.

Our results showed that both voiced–voiceless contrast of consonants and the position of the tongue when producing vowels contributed to the sound–shape correspondence dominantly and equally (see also D’Onofrio, 2014). Compared to these two factors, the stop–continuant contrast of consonants was a weaker predictor. Unlike other studies using categorical predictors, such as phonetic features or letters, we used normalized proportions along contrasting dimensions as the predictors. Such predictors implied the existence of both contrastive and continuous markings in crossmodal correspondences. In other words, a higher proportion of back vowels and voiced and stop consonants would map to more rounded shapes. Figure 3 demonstrates the coordinates of the tested pseudowords on the axes of the voice–voiceless and stop–continuant dimensions, and the darkness of the words and the outline of circles represents the proportion of back vowels (darker: more back vowel; brighter: more front vowel). The filled circles represent the consensual level of matching (red: rounded shapes; blue: angular shapes, based on the results in Figure 2). Three predictors, the position of the vowel, and voiced–voiceless and stop–continuant contrasts of the consonants, can be easily applied to sound–shape correspondences especially when using realistic pseudowords.

The sound–shape matching results showed that three pseudowords (eposomo, kanktil, and keppick) did not consensually match to the expected shape according to Westbury et al.’s (2018) results. This might have been due to methodological differences. In Westbury et al.'s study, the concept of sharp/round was investigated by presenting the written word on a monitor, and the tested pseudowords were presented both auditorily and visually. However, we presented two visual shapes classified into angular and rounded contrasting dimensions, where the tested pseudowords were presented merely auditorily. We investigated only the association between sound and shape and excluded/reduced other modulating cues (e.g., the length of words, orthographic influences among literate participants) to such correspondences (Brackbill & Little, 1957; Cuskley et al., 2017). The other possibility explaining the differences in the outcome may have been language experience. All of our participants were native Mandarin speakers who have also learned English for at least ten years (based on the education system). Although the bouba–kiki effect is believed to be universal (Ramachandran & Hubbard, 2005), recent studies have also shown subtle differences in sound–shape correspondences between people from eastern and western cultures when the visual patterns were manipulated systematically (Chen et al., 2016). Furthermore, if the pseudowords did not follow phonetic legality (wordiness) in the participant's language, the matching would be less predictable (Styles & Gawne, 2017). Those substantial differences across countries/cultures suggested that the bouba–kiki effect is sensitive to different perceptual styles and language experience to a certain extent (e.g., Chen et al., 2016; Rogers & Ross, 1975; Shang & Styles, 2017).

### Conclusion

We verified sound–shape correspondences at the phonetic level by demonstrating systematic mapping between the contrasting dimensions in vowels and consonants and the rounded-angular dimension in visual shapes. The sound–shape mappings were predictable using contrasting in position when producing vowels (back, central and front) and voiced–voiceless and stop–continuant dimensions of consonants. The English phonemes and visual shapes used in the current study provide useful tools to examine the universality of crossmodal correspondences in future studies. Our results also showed that not all of the pseudowords had consensus matching among different methods and different native languages (cf. Westbury et al., 2018), and thus future research can further explore the influence of language experience in sound–shape correspondences.

## Notes

1. We also added the participant's gender as one of the predicting factors and the results showed no effects on gender (*β* = −0.029, *p* = .76). This factor was therefore removed in the final analysis.

2. In order to verify how the matchings to visual pattern pairs differed from each other, we also conducted the logistic regression that treats the visual pattern as one of the predictive fixed factors and used different visual pattern pairs as baseline. Note that this factor was categorical because we did not manipulate the amplitude and/or frequency systematically. The coefficient values for visual pair comparisons showed statistical differences (e.g., the difference between (B) pair and (C) pair) for all pair comparisons (all *p* < .01), except the visual pattern comparison between (A) pair and (C) pair (*β* = 0.12, *p* = .09). Nevertheless, the coefficient values for vowels and consonants were similar to those values that treated visual pattern as a random factor, suggesting that the modulations of the vowels, and voiced-voiceless and stop-continuant consonants were reliable in the sound–shape correspondences. Therefore, in the main text, we kept the results which treated visual patterns as a random factor.
